# Toward Large-Scale Production of Oxidized Graphene

**DOI:** 10.3390/nano10020279

**Published:** 2020-02-06

**Authors:** Talia Tene, Gabriela Tubon Usca, Marco Guevara, Raul Molina, Francesco Veltri, Melvin Arias, Lorenzo S. Caputi, Cristian Vacacela Gomez

**Affiliations:** 1 Department of Chemistry and Exact Sciences, Universidad Técnica Particular de Loja, Loja EC-110160, Ecuador; tbtene@utpl.edu.ec; 2 Faculty of Science & Mechanical Engineering, Escuela Superior Politécnica de Chimborazo, Riobamba EC-060155, Ecuador; gabriela.tubon@espoch.edu.ec (G.T.U.); marco.guevara@espoch.edu.ec (M.G.); 3 UNICARIBE Research Center, University of Calabria, I-87036 Rende (CS), Italy; francesco.veltri@unical.it (F.V.); melvin.arias@intec.edu.do (M.A.); lorenzo.caputi@fis.unical.it (L.S.C.); 4 GraphenTech NL, Olympiaweg 28A, 3077AL Rotterdam, The Netherlands; rm@redecua.com; 5 Surface Nanoscience Group, Department of Physics, University of Calabria, Via P. Bucci, Cubo 33C, I-87036 Rende, Italy; 6 Instituto Tecnológico de Santo Domingo, Area de Ciencias Básicas y Ambientales, Av. Los Próceres, Santo Domingo 10602, Dominican Republic; 7 CompNano, Yachay Tech University, School of Physical Sciences and Nanotechnology, Urcuquí EC-100119, Ecuador

**Keywords:** graphene, oxidized graphene, citric acid, large-scale production

## Abstract

The oxidative exfoliation of graphite is a promising approach to the large-scale production of graphene. Conventional oxidation of graphite essentially facilitates the exfoliation process; however, the oxidation procedure releases toxic gases and requires extensive, time-consuming steps of washing and reduction to convert exfoliated graphene oxide (GO) into reduced graphene oxide (rGO). Although toxic gases can be controlled by modifying chemical reactions, filtration, dialysis, and extensive sonication are unfavorable for large-scale production. Here, we report a complete, scalable, and green synthesis of GO, without NaNO_3_, followed by reduction with citric acid (CA). This approach eliminates the generation of toxic gases, simplifies the washing steps, and reduces the time required to prepare rGO. To validate the proposed method, we present spectroscopical and morphological studies, using energy-dispersive X-ray spectroscopy (EDS), UV-visible spectroscopy, infrared spectroscopy, Raman spectroscopy, scanning electron microscopy (SEM), and transmission electron microscopy (TEM). Thermal gravimetric analysis (TGA) is used to analyze the thermal properties of GO and rGO. This eco-friendly method proposes a complete guideline protocol toward large-scale production of oxidized graphene, with potential applications in supercapacitors, fuel cells, composites, batteries, and biosensors.

## 1. Introduction

Graphene, a two-dimensional (2D) single atomic layer of sp^2^ carbon atoms [1], has attracted intense research interest, owing to its high surface area, excellent thermal and electrical properties, extended charge carrier mobility, high elastic behavior, and optical tunability [1,2,3,4,5]. Transferable single-layer graphene was first obtained by mechanical exfoliation [6] of bulk graphite and by chemical vapor deposition [7]. Although these routes produce high-quality graphene, they can be less effective for large-scale manufacturing. The major obstacle to achieving single-layer or few-layer graphene is the interlayer van der Waals interaction [8]. Keeping this consideration in mind, the most common approaches to the large-scale synthesis of graphene rely on liquid-phase exfoliation [9,10] or oxidation–reduction of graphite [11,12]. The key differences between these methods are the yield and the defect content of their products. Defect-free graphene is mostly preferred for electronic applications [13], whereas in some other cases, defects and imperfections in graphene are less relevant and desirable [14,15,16]. Some achievements have improved liquid–exfoliation processes [17,18,19] to obtain large quantities of graphene. However, the obtained graphene mainly has a hydrophobic feature and a small lateral size. Thus, the oxidation–reduction of graphite is a more practical method for bulk-scale graphene materials in immediate applications—e.g., to produce colloidal suspensions for versatile processing of thin films and composites [20,21].

Typically, the oxidation–reduction of graphite uses strong oxidizing agents to yield graphene oxide (GO), an insulating carbon material [22]. To date, the basic structure of GO remains ambiguous due to the vast number of functional groups and the entailed isomeric possibilities. It is well-known that, depending on the degree of oxidation, the aromatic lattice of graphene exhibits different oxygen functional groups, such as hydroxyl, epoxy, carboxyl, and carbonyl groups [22,23,24], which increase the interlayer spacing from 0.33 nm (graphite) to 0.87 nm (GO) [25]. At a low degree of oxidation, hydroxyl groups can chemically turn into epoxide groups and vice versa, allowing for the use of GO for specific technological applications [24]. In addition, the GO structure reveals a mixed sp^2^−sp^3^ hybridization as a direct consequence of the degree of oxidation acquired during the oxidation process [26] (see, e.g., Brodie [27], Staudenmaier [28], Hummer [29], Marcano [30], and Chen [31]). Hummer et al. reported the method most used nowadays: the oxidation of graphite is realized by treatment with KMnO_4_ and NaNO_3_ in concentrated H_2_SO_4_. This procedure involves the generation of toxic gases, such as NO_2_ and N_2_O_4_, and the residual Na^+^ and NO_3_^−^ ions are difficult to remove during the processes of synthesizing and purifying GO [29,30,31]. While the induced oxygen functional groups increase the distance between graphitic layers (weakening the van der Waals forces and facilitating the preparation of GO), the removal of oxidizing agents and other impurities from GO is obtained through conventional methods such as filtration, dialysis, or sonication, making the Hummer method unsuitable for scaling at an industrial level.

To circumvent these environmental and technical issues, Chen et al. [31] have improved the Hummer method by excluding NaNO_3_. The authors successfully demonstrated that this modification does not decrease the yield of the product, but reduces toxic gas evolution and makes the disposal of wastewater more straightforward due to the absence of Na^+^ and NO_3_^−^ ions [31]. Nevertheless, the obtained graphite oxide needs to be filtered and purified by dialysis for one week to remove metal ions. Additionally, the resulting graphite oxide needs to be diluted in 1.2 L of water, stirred overnight, and sonicated for 30 min to give, finally, GO powder. For large-scale applications of graphene, the filtration and dialysis are a bottleneck. These steps are time-consuming, since graphite oxide particles (especially exfoliated ones) rapidly block the filter and membrane pores. Also, the reduction process adds yet another step to the synthesis procedure, prolonging the overall production time [32]. Hence, there is a great challenge to develop a green, low-cost, and efficient reduction method.

To support this direction, Abdolhosseinzadeh et al. [33] reported a sonication-assisted oxidation method for large-scale production of GO followed by the reduction of GO with ascorbic acid before washing steps, which relatively decreased the production time of reduced graphene oxide (rGO). This method proposes the preparation of GO by the Hummer method, using a stirring–sonication process of 5 min 12 times, and an extra ultrasonication for 2 h prior to reduction. After reduction, the obtained material is sonicated for 1 h and filtrated to obtain rGO powder. Nevertheless, the procedures involving extended sonication have limited scalability using an extensive sonication time, which may induce basal/edge defects, a small lateral size, and high-cost production [34]. Recently, Garino et al. [35] reported a microwave-assisted fast synthesis of rGO co-doped with manganese and nitrogen through a green approach. The co-doping with nitrogen and manganese occurs while reducing GO.

To the best of our knowledge, a reliable method with the capability to supply the large demand for pristine GO and (undoped) rGO by using a green approach, a short-sonication time, and viable washing steps is still lacking. In this communication, such a method is presented, considering the environmental problem and technical limitations. We successfully demonstrate, through several spectroscopy and morphological studies, an eco-friendly and complete guideline protocol for large-scale production of GO by excluding NaNO_3_ [31] and adding citric acid (CA) after a simple washing process. The advantages of this method, with its simple protocol, preserved yield, and lack of extensive sonication and toxic gas evolution during preparation of GO, make it attractive for preparing rGO on a large scale, with promising applications in composites, energy storage, and reinforcement, where a large quantity of oxidized graphene is needed [36].

## 2. Materials and Methods

**Figure 1:**
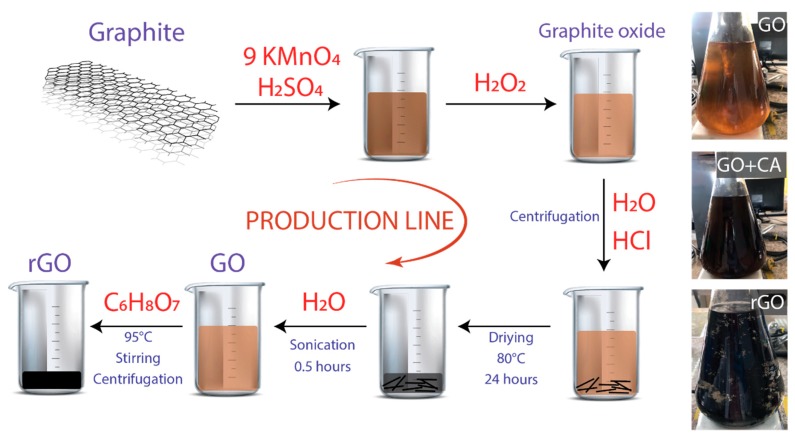
Schematic representation of the procedure. The starting material is graphite powder. Inset: optical images of graphene oxide (GO), GO plus citric acid (CA), and reduced graphene oxide (rGO).

Graphite powder (<150 μm, 99.99%), sulfuric acid (H_2_SO_4,_ ACS reagent, 95.0–98.0%), potassium permanganate (KMnO_4,_ ACS reagent, ≥99.0%), hydrochloric acid (HCl, ACS reagent, 37%), and citric acid (C_6_H_8_O_7_, ACS reagent, ≥99.5%) were obtained from Sigma Aldrich. All chemicals were used as received, without further purification. Figure 1 shows the schematic route of the production line for GO and rGO.

### 2.1. Synthesis of GO

It is important to stress once more that the synthesis of GO is based on the approach of Ref. [31]. However, some obvious modifications were developed to make the process as simple as possible. In the typical experiment, 3.0 g of graphite powder was added to 70 mL of concentrated H_2_SO_4_ while stirring in an ice-water bath. Then, 9.0 g of KMnO_4_ were gradually added, maintaining the temperature of the suspension to be lower than 20 °C. The resulting mixture was transferred to a 50 °C oil bath and vigorously agitated for about 0.5 h. Distilled water (150 mL) was added, and the solution was stirred for 20 min at ~90 °C. Additionally, 500 mL distilled water was added, followed by a slow addition of 15 mL of hydrogen peroxide (H_2_O_2,_ 30%, Merk) and stirred up to turn the color of the solution from dark brown to yellowish. The resulting graphite oxide suspension was washed with 1:10 HCl solution and distilled water several times, each for 10 min by means of centrifugation at 3000 rpm until the pH was at ~6. The precipitated material was dried at 80 °C for 24 h.

### 2.2. Synthesis of rGO

Initially, 50 mg of resulting solid was dispersed in 500 mL of distilled water by sonication for 0.5 h, employing an ultrasonic bath (Branson 2510 Ultrasonic Cleaner, Framingham, MA, USA) in continuous operation. Bath sonication is preferred to tip sonication because the direct positioning of the tip in the medium results in strong material fragmentation [37]. The obtained suspension was centrifugated at 1000 rpm for 0.5 h and divided into two equal parts: one to obtain a homogenous GO suspension (Figure 1, optical images), and the other was further processed for preparing rGO. Under vigorous agitation, 250 mg CA was slowly added in the remaining suspension. Different reduction times were tested (from 0.5 to 3 h), setting the reduction temperature at 95 °C. To remove excess CA, the resultant black precipitates were washed with distilled water by centrifugation at 3000 rpm for 0.5 h. Finally, the precipitated material was dried at 80 °C overnight to obtain rGO powder.

### 2.3. Characterization

The characteristic absorption spectra of GO and rGO were recorded using UV-vis spectroscopy (Thermo Scientific, Evolution 220, Waltham, MA, USA). Infrared spectra were collected using a Fourier transform infrared spectrometer Jasco FT/IR-4000 (Easton, MD, USA). The thermal stability of GO and rGO was investigated using thermogravimetric analysis (TGA, PerkinElmer simultaneous thermal analyzer, STA 6000, Waltham, MA, USA). The structure and surface morphology of the obtained materials were taken out on a transmission electron microscope (TEM, JEM 1400 Plus, JEOL, Musashino, Akishima, Tokyo, Japan) operating at 80 kV, and a scanning electron microscope (SEM, JSM-IT100 InTouchScope, JEOL, Musashino, Akishima, Tokyo, Japan) equipped with a JEOL-made dispersive X-ray spectrometer (EDS) with the accelerating voltage of 15 kV. Raman spectra were obtained using a Jasco NRS-500 (Easton, MD, USA) spectrometer with a 532 nm laser wavelength (0.3 mW, 100X objective). SEM samples were prepared by drop casting on aluminum substrates and dried at 80 °C for 2 h. Similarly, TEM and Raman samples were prepared by drop casting onto formvar-coated copper grids and glass substrates, respectively.

## 3. Results and Discussion

The main intent of this work is to propose a general eco-friendly approach to large-scale production of oxidized graphene. In this sense, we have selected CA because it is a green and low-cost alternative for conventional reducing agents of GO [38] (e.g., hydrazine hydrate, hydroquinone, sodium borohydride, and hydrogen sulfide), which can acceptably reduce the obtained GO as discussed in the following. Furthermore, CA together with hydrazine hydrate [39] or alkali hydroxide [40] has been used to prepare reduced graphene oxide or graphene oxide quantum dots.

After treatment with CA, the yellowish GO solution turned black, and a stable dispersion of rGO flakes could be detected by the naked eye, as displayed in Figure 1 (optical images). The optical change of GO (yellowish suspension) to rGO (black suspension) strongly confirms the reduction of oxygen functional groups (Figure S1). GO and rGO flakes are easy to precipitate as time increases. However, the stability of the GO suspension was higher than that of the rGO suspension within 72 h. In general, graphene tends to aggregate and precipitate in aqueous media due to its hydrophobicity and the strong π−π interactions between graphene layers. Therefore, the observed sedimentation supports the formation of rGO [41].

**Figure 2:**
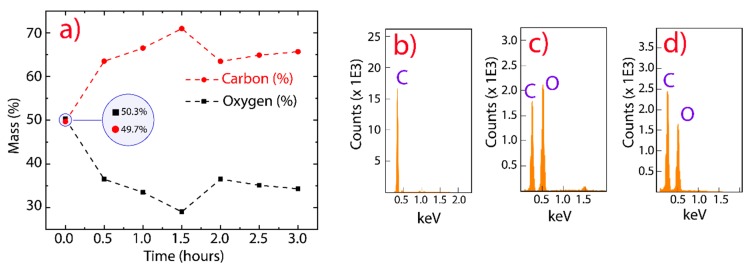
EDS analysis. (**a**) Elemental compositions (%) as a function of the reduction time by using citric acid. (**b**–**d**) EDS spectra of graphite, GO, and rGO, respectively.

In order to investigate the elemental composition of GO and rGO, we performed EDS measurements, controlling the reduction temperature and testing different reduction times (Figure 2a). As the EDS technique depends on the bombarded region, the investigated area was large enough to ensure the reliability of the results. As the interaction time increased between GO and CA, the reduction effectivity increased from 0.5 to 1.5 h; however, a slight oxidation was observed after 2 h. This result could be attributed to a partial oxygen functionalization from water molecules because the reduction was carried out at 95 °C. In particular, the observed reduction of oxygen element was about 7% at 0.5 h and 13% at 1.5 h. We focused on the reduction at 0.5 h to optimize the production process, avoid a probable instable reduction, and preserve a good dispersibility in water. The latter is an important feature that may be exploited in antibacterial and cytotoxic applications [42]. Figure 2b–d (and Figure S2) reveal that pristine graphite, GO, and rGO are mainly composed of carbon and oxygen elements in the accelerating-voltage window from 0 to 5 keV. 

**Figure 3:**
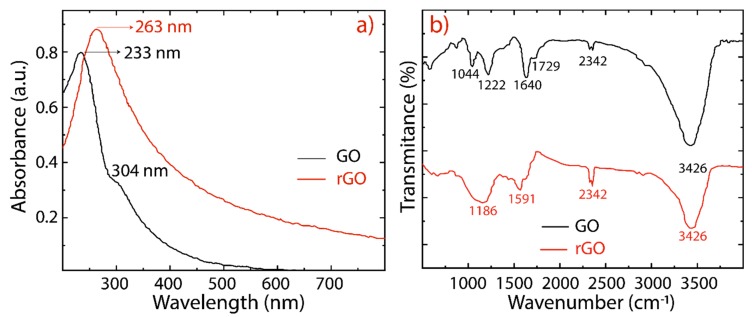
(**a**) UV-visible spectra recorded in aqueous solutions at 0.1 mg/mL of GO (black line) and rGO (red line). (**b**) Fourier transform infrared spectra of GO (black line) and rGO (red line).

The reduction of GO into rGO was further confirmed by UV-vis absorption spectroscopy and the observed results are depicted in Figure 3a (and Figure S3). GO exhibits two absorption peaks (black line) at 233 nm, and a shoulder peak at 304 nm, which are attributed to the π−π* transitions of C−C, and n−π* transitions of C=O bonds, respectively. The absorption peak observed at 233 nm of GO has been red shifted to 263 nm for rGO (red line), suggesting that the electronic conjugation of the aromatic structure might be restored. The overall features of these spectra and their absorption peaks are similar to those of GO and rGO reported in the literature [30,31,43]. Most importantly, the rGO spectra, considering different reduction times, are featureless in the visible region, as expected for graphene (Figure S3).

The oxygen-containing functional groups are confirmed in the FTIR spectra given in Figure 3b. As is well-known, the hydroxyl and epoxide groups attached to the basal plane of the graphene and carboxyl and carbonyl groups located at the edges are the dominant functional groups [24]. The following characteristic functional groups are detected: C−O−C (1044 cm^−1^), C−O (1222 cm^−1^), C=C (1644 cm^−1^), and C=O (1729 cm^−1^). The broad peak observed at 3426 cm^−1^ is due to the hydroxyl groups (O−H) and adsorbed water molecules between GO sheets. This hydrophilic feature provides GO sheets with a good dispersibility in water. After reduction, the prominent peaks in the GO spectrum result are significantly attenuated and weakened in the rGO spectrum, confirming the removal of oxygen functional groups. The band observed at 2360 cm^−1^ in GO and rGO is assigned to the CO_2_ stretching vibration [44].

**Figure 4:**
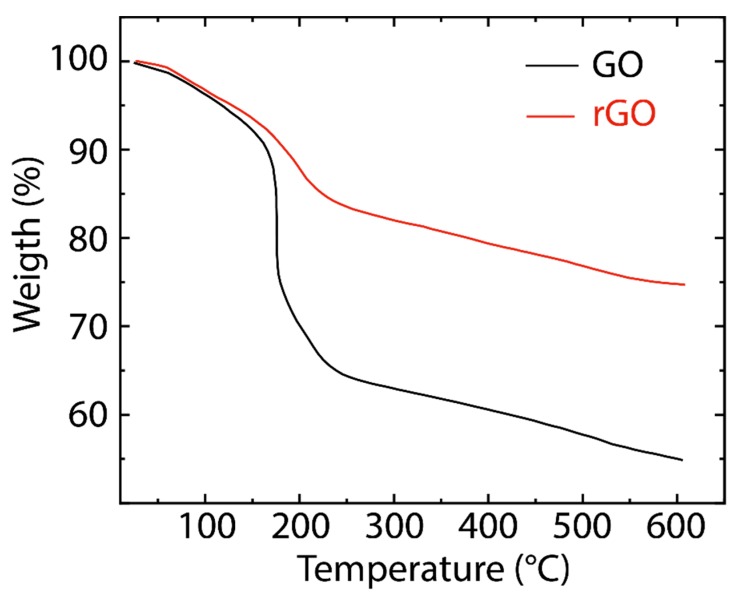
Thermogravimetric analysis (TGA) of GO (black line) and rGO (red line).

Figure 4 presents the TGA curves of GO and rGO. In GO, the minimal weight loss (~5%) before 100 °C is attributed to the loss of physisorbed water molecules [44]. The significant weight loss (~25%) in the range of 200–250 °C and weaker mass loss (~10%) in the range of 250–600 °C are attributed to the pyrolysis of less stable (CO, CO_2,_ and H_2_O) and more stable oxygenated functional groups, respectively [45]. On the other hand, rGO reveals a higher thermal stability compared to GO due to the graphitization and promoted van der Waals forces between the layers through the removal of oxygen functional groups [44]. TGA curves of both GO and rGO reflect a similar trend, suggesting their close contents of oxygenated groups, as the FTIR data indicated.

**Figure 5:**
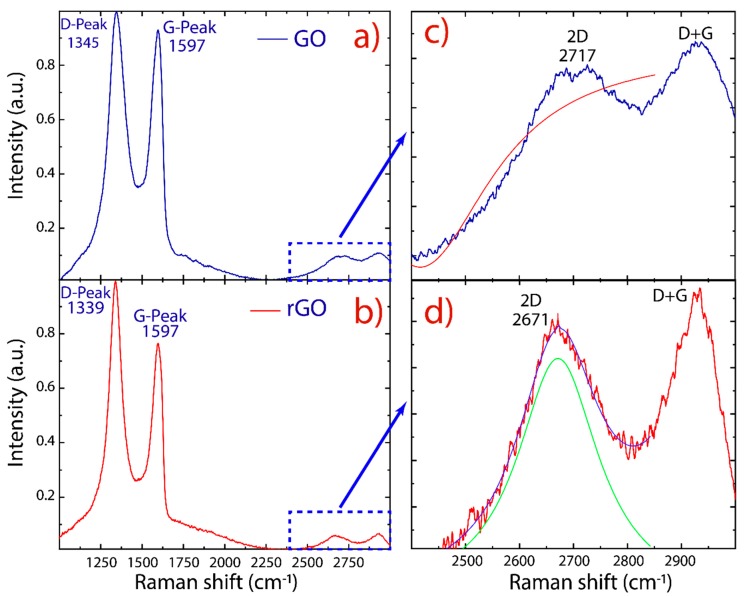
Raman spectra recorded using 532 nm laser excitations. (**a**) GO and (**b**) rGO. (**c**,**d**) Fitting of the 2D peaks with Lorentzian functions. The intensity was normalized by the D peak.

Raman spectroscopy was used to examine structural changes during the oxidation and reduction processes. The Raman spectra of GO and rGO are reported in Figure 5. As is typical for oxidized graphene, two prominent peaks are detected: i.e., the D peak at ~1345 cm^−1^ and the G peak at ~1597 cm^−1^. Other less intense peaks also have been detected: the 2D peak at ~2700 cm^−1^ and the D+G peak at ~2920 cm^−1^. The D and D+G (the combination of D and G bands) peaks are ascribed to structural imperfections of GO and rGO (vacancies, heptagon/pentagon rings, the edge effect [46,47,48]) while the G peak is related to the in-plane bond-stretching motion of sp^2^-hybridized carbon atoms in the graphene/graphite lattice. On the other hand, the 2D peak is regularly used to estimate the number of layers in obtained graphene.

The peak ratio between the intensity of D and G peaks (*I_D_/I_G_*) is a common index for the density of defects on GO and rGO. As shown in earlier studies [48,49,50], it was found that the intensity ratio of rGO (~1.29) is larger than that of GO (~1.09), indicating that the size of the graphene-like domains is diminished after exposure to reduction and sonication, but they are more numerous in number [48]. Interestingly enough, the D and G peaks of rGO are more separated from each other and are distinctly different from GO, where the D and G peaks are broad and overlap: i.e., the increased intensity of the D peak to G peak is due to the elimination of defects [33], suggesting that rGO did not undergo severe structural disruption compared to GO.

Unlike the 2D peak of monolayer graphene, which has high intensity, the 2D peak of GO and rGO is complex in shape and evolves with the number of layers [46]. The presence of an intense D peak and a weak 2D peak is a general feature of graphene produced by oxidation–reduction-based methods. However, the intensity of the 2D peak also depends on the excitation laser frequency, and therefore cannot be solely relied upon [51]. In this context, we used the full width at half maximum (FWHM) to evaluate the 2D peak. By fitting the 2D peak with Lorentzian functions, the 2D peak (~2671 cm^−1^) of rGO is shifted with respect to the 2D peak (~2717 cm^−1^) of GO. The latter was impossible to be fitted, and the maximum intensity value was removed from the data (Figure 5c). The respective FWHM of rGO was found to be 183.6 ± 7.0 cm^−1^. Furthermore, the 2D peak became more symmetrical in rGO, indicating the presence of few-layer-reduced graphene oxide.

**Figure 6:**
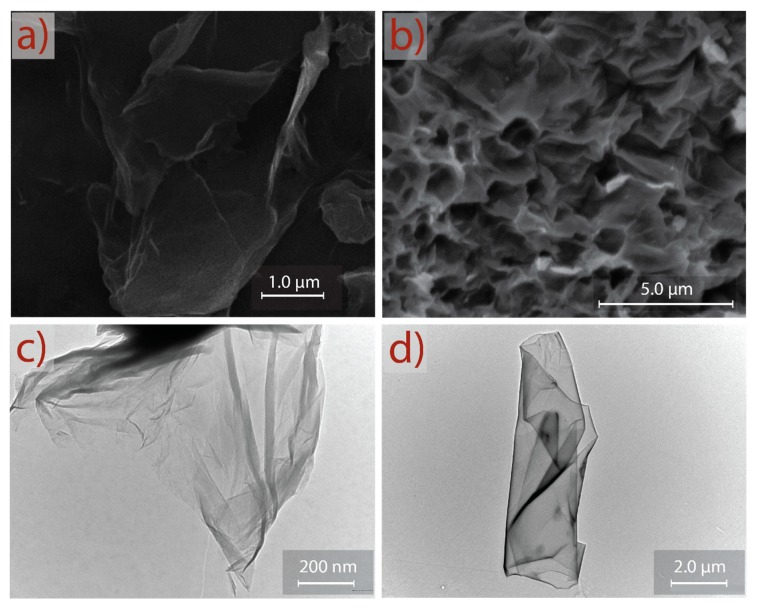
SEM morphology of (**a**) GO and (**b**) rGO. TEM images of (**c**) GO and (**d**) rGO.

SEM and TEM micrographs of GO and rGO are shown in Figure 6. The observed morphology of GO (Figure 6a) consists of randomly aggregated, transparent, and flake-like sheets with wrinkles and folds on the surface of GO, as well as face-to-face stacking of sheets. The surface morphology of rGO (Figure 6b) shows a significant difference compared to GO. A porous surface (after reduction and sonication) is discovered, and rGO shows a highly distorted surface that can prevent face-to-face stacking of the graphene layers by the formation of mesopores and macropores. The semitransparent layers in GO and rGO seem to be free of impurities, which can be interpreted as an important result to prove that extensive or time-consuming washing processes (e.g., filtration, dialysis, ultrasonication, sonication) can be evaded.

This asseveration is supported by TEM analysis. Figure 6c shows a representative TEM image of GO. A transparent and thin nanosheet with some wrinkles and folds on the surface and edges is observed for GO. At first approximation, the GO nanosheet analyzed in this work appears to be very similar to the GO samples prepared by conventional oxidation–reduction-based methods; however, a clear difference is observed—i.e., the GO prepared by commonly used methods are mostly folded or have a folded edge due to a strong oxidation produced in the presence of NaNO_3_ [29,30]_._ Figure 6d presents a well-defined rGO nanosheet obtained after reduction, sonication, and washing. The observed regular surface suggests that the proposed method does not induce critical basal-plane damage during oxidation or reduction processes. These results confirm an eco-friendly and complete guideline protocol that can be implemented for the large-scale production of oxidized graphene through a reliable approach, short-sonication time, and simple washing steps.

## 4. Conclusions

In summary, we have established a complete and green protocol for the preparation of GO and rGO by excluding NaNO_3_ in the oxidation and adding CA in the reduction, which demonstrated to be an effective and inexpensive reducing agent for GO. EDS measurements showed a reduction effectivity from 0.5 to 1.5 h, while GO is in contact with CA under vigorous agitation. UV-visible spectra confirmed the reduction of GO into rGO. FTIR analysis demonstrated the presence of oxygen functional groups, which decreased in intensity after reduction. The thermal stability of rGO was found to be higher compared to GO. Raman spectra showed a high intensity of the D peak in GO, indicating the presence of basal/edge defects; however, the characteristic 2D peak of graphene arose in rGO. SEM measurements showed that the semitransparent layers were relatively free of impurities. TEM analysis presented similar features. However, rGO exhibited a more regular, transparent, and thin nanosheet. The proposed method prevents toxic gas evolution, prolonged sonication, and complicated washing steps, making it effective at preparing oxidized graphene on a large scale.

## Supplementary Materials

The following are available online at https://www.mdpi.com/2079-4991/10/2/279/s1, Figure S1. Optical images of rGO (black) suspensions. To obtain suspensions from rGO powder, the reduction of GO with CA was carried out considering three different times (0.5, 1.0, 1.5 h) with the procedure outlined in the main text. Then, 10 mg of rGO powder were added into 10 mL distilled water and sonicated for 30 min. The obtained suspensions were centrifugated for 30 min at 3000 rpm. Figure S2. EDS spectra of GO and rGO considering different reduction times (0.5 h, 1.0 h and 1.5 h). The carbon and oxygen elements are predominant in the range from 0 to 5 keV. Figure S3. UV-visible spectra recorded in aqueous solutions at 0.1 mg/mL of (a) GO and (b–d) rGO considering different reduction times (0.5 h, 1.0 h and 1.5 h). The intensity was normalized by the predominant peak.
