# Supplementary material for: Toward Large-Scale Production of Oxidized Graphene
Source: Nanomaterials (Basel). 2020 Feb 6;10(2):279. doi: 10.3390/nano10020279 (PMC7075232; doi:10.3390/nano10020279)
Supplement: Supplementary file 1 [file nanomaterials-10-00279-s001.pdf]

Toward Large-Scale Production of Oxidized Graphene

Talia Tene, Gabriela Tubon Usca, Marco Guevara, Raul Molina, Francesco Veltri, Melvin Arias, Lorenzo S. Caputi, and Cristian Vacacela Gomez

Supplementary Figures

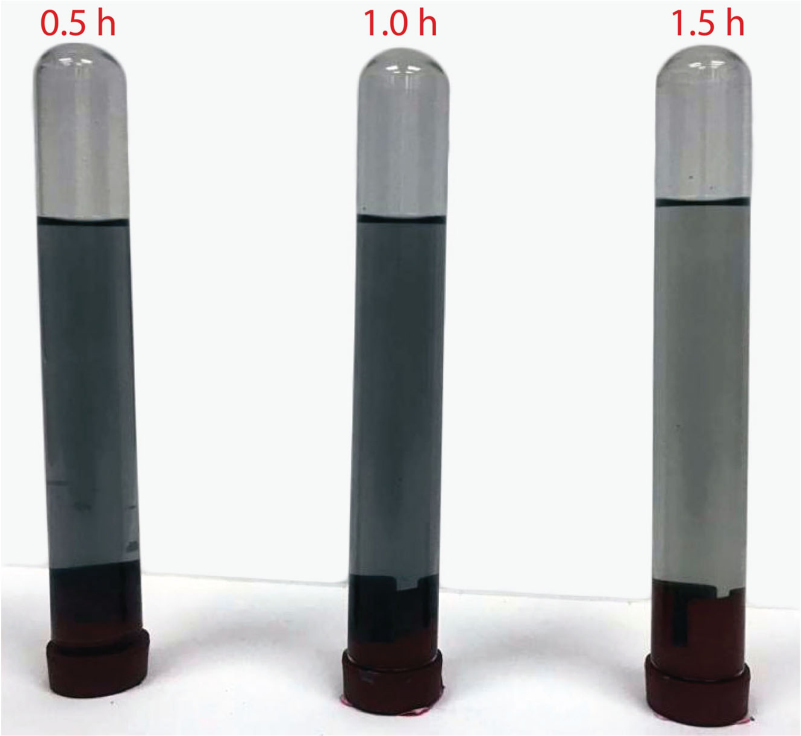

Figure S1. Optical images of rGO (black) suspensions. To obtain suspensions from rGO powder, the reduction of GO with CA was carried out considering three different times (0.5, 1.0, 1.5 h) with the procedure outlined in the main text. Then, 10 mg of rGO powder were added into 10 ml distilled water and sonicated for 30 min. The obtained suspensions were centrifugated for 30 min at 3000 rpm.

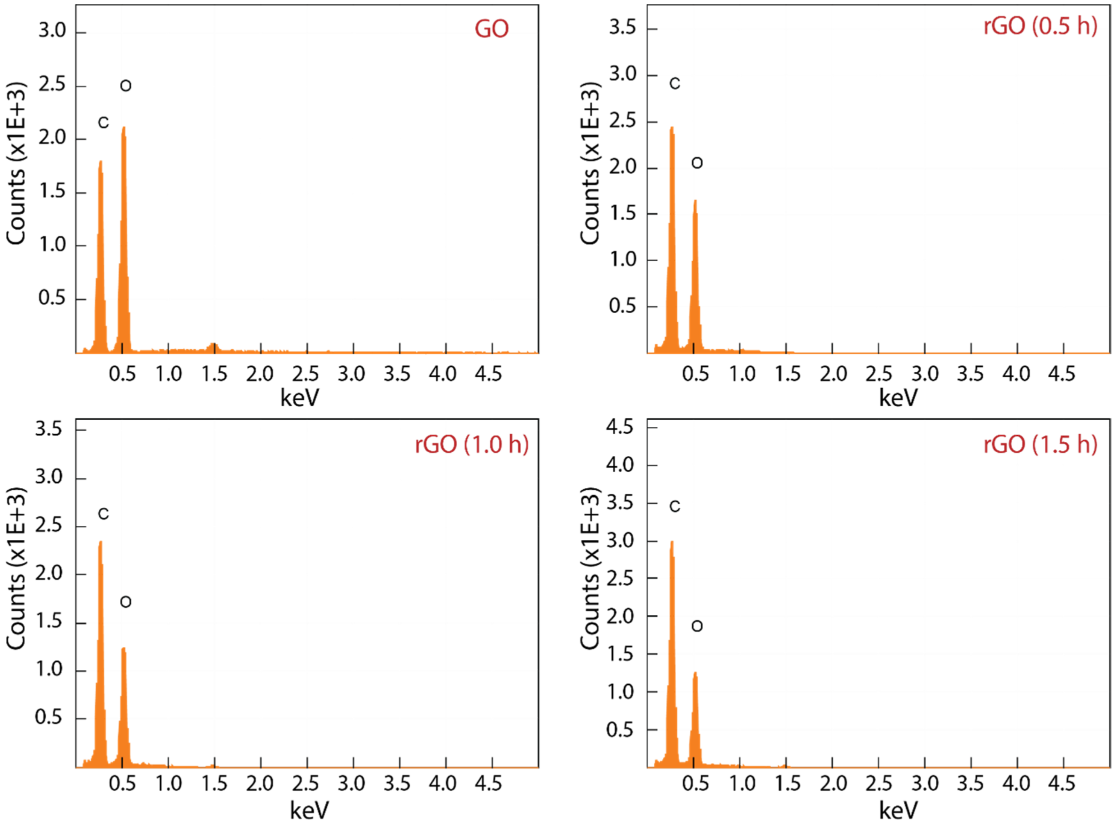

Figure S2. EDS spectra of GO and rGO considering different reduction times (0.5 h, 1.0 h and 1.5 h). The carbon and oxygen elements are predominant in the range from 0 to 5 keV.

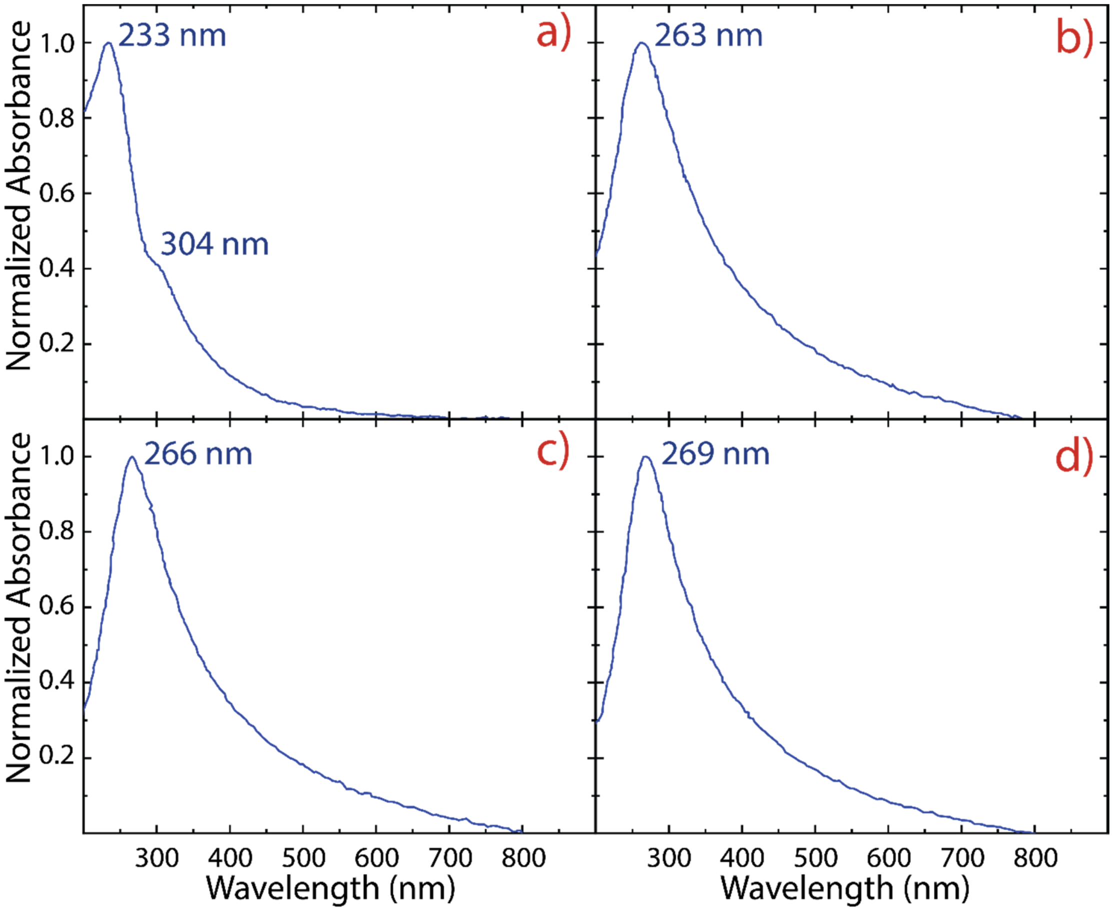

Figure S3. UV-visible spectra recorded in aqueous solutions at 0.1 mg/mL of (a) GO and (b-d) rGO considering different reduction times (0.5 h, 1.0 h and 1.5 h). The intensity was normalized by the predominant peak.
